# Supplementary material for: Vectorial application for the illustration of archaeological lithic artefacts using the “Stone Tools Illustrations with Vector Art” (STIVA) Method
Source: PLoS One. 2021 May 11;16(5):e0251466. doi: 10.1371/journal.pone.0251466 (PMC8112888; doi:10.1371/journal.pone.0251466)
Supplement: S1 File — Also available on protocols.io. (PDF) [file pone.0251466.s001.pdf]

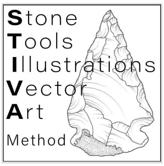

Version 2

Apr 19, 2021

Stone Tools Illustrations with Vector Art: The 'STIVA' Method V.2

Jacopo Niccolo Cerasoni[1]

[1]Pan African Evolution Research Group, Max Planck Institute for the Science of Human History, Jena, Germany

1 Works for me dx.doi.org/10.17504/protocols.io.bubqnsmw

Jacopo Niccolo Cerasoni

ABSTRACT

Lithic illustrations are often used in scientific publications to efficiently communicate the technological and morphological characteristics of stone tools. They offer invaluable information and insights not only on how stone raw materials were transformed into their final form, but also on the individuals that made them. Here, the "Stone Tools Illustrations with Vector Art" (STIVA) Method is presented, which involves the illustration of lithic artefacts using vectorial graphics software (Adobe® Illustrator®). This protocol follows an optimised step-by-step method, presenting ten major sections that constitute the creation of a lithic illustration: photography, vectorial software configuration, scale, outline, scar borders, ripples, cortex, symbols, composition, and export. This method has been developed to allow researchers, students and educators to create clear and competent illustrations for any application, from scientific publications to public outreach.

DOI

dx.doi.org/10.17504/protocols.io.bubqnsmw

PROTOCOL CITATION

Jacopo Niccolo Cerasoni 2021. Stone Tools Illustrations with Vector Art: The 'STIVA' Method. **protocols.io**
https://dx.doi.org/10.17504/protocols.io.bubqnsmw
Version created by Jacopo Niccolo Cerasoni

WHAT'S NEW

1. Steps 22 and 23 of v.1 have been merged. 2. Step 26 (step 27 in v.1) has been updated to give clearer instructions on ripple mark cutting.

KEYWORDS

Stone Tools, Lithics, Illustration, Archaeology, Anthropology, Prehistory, Material Culture, Technical Drawing

LICENSE

This is an open access protocol distributed under the terms of the Creative Commons Attribution License, which permits unrestricted use, distribution, and reproduction in any medium, provided the original author and source are credited

CREATED

Apr 19, 2021

LAST MODIFIED

Apr 19, 2021

PROTOCOL INTEGER ID

49232

Citation: Jacopo Niccolo Cerasoni (04/19/2021). Stone Tools Illustrations with Vector Art: The 'STIVA' Method. https://dx.doi.org/10.17504/protocols.io.bubqnsmw

This is an open access protocol distributed under the terms of the **Creative Commons Attribution License** (https://creativecommons.org/licenses/by/4.0/), which permits unrestricted use, distribution, and reproduction in any medium, provided the original author and source are credited

GUIDELINES

In the following protocol, typographical emphases and brackets have been used for software and in-text references.
They are: (1) **software features** and **tools** written in **bold**, e.g. **Curvature Tool**; (2) "vector layers" in "quotation marks", e.g. "outline"; (3) references to *steps* of the protocol in *italics,* e.g. step *21*; (4) <keyboard buttons> in <angle markers>, e.g. <Canc> or <Del>; and (5) computational workflows linked by angle markers (>), e.g. <right click> > **Arrange** > **Bring to Back.**

Please note: This protocol has been developed for Adobe® Illustrator®, being specifically developed using Adobe® Illustrator® 2020. Nevertheless, previous Adobe® Illustrator® versions and other vector graphics softwares can be used (e.g. CorelDRAW® and Inkscape®). If previous versions or different vectorial softwares are being used, some functionalities will have to be accessed and applied differently.

MATERIALS TEXT

List of materials needed:

• Camera and tripod (ideally with macro lens; 90mm to 105mm focal length)
• Light box (optional)
• Measuring scale
• White or grey cardstock/velvet cloth
• Vectorial graphics software (Adobe® Illustrator® 2020)
• Graphic tablet (optional, although it is advisable to use for better efficiency and productivity)

Photograph Artefact

1 Lock camera on a tripod (ideally use a macro lens with a focal length of between 90mm to 105mm) and place in light box (if available).

2 Place artefact flat onto the workspace.

2.1 If the artefact does not sit flat due to its irregular shape, use an appropriate amount of modelling clay wrapped in plastic wrap so to create a modellable support surface.

3 With lamps or other light-sources set up direct and suffused lighting from one or more sides and the top. Scar shadows should appear. Try to prevent major shadowing on the background surface.

4 Take several photographs of the artefact, moving the lighting at different angles and heights so to create different shadow orientations.

5 Select the preferred photograph, which should have the highest degree of visibility of the single scars and ripples.

6 Repeat processes *2-5* for the remaining sides of the artefacts which you want to illustrate (e.g. ventral face, dorsal face, profile, platform).

7 Choose the photographs that will be used in the next steps, and save them in a separate folder. Do not delete the remaining photographs.

Citation: Jacopo Niccolo Cerasoni (04/19/2021). Stone Tools Illustrations with Vector Art: The 'STIVA' Method. https://dx.doi.org/10.17504/protocols.io.bubqnsmw

This is an open access protocol distributed under the terms of the **Creative Commons Attribution License** (https://creativecommons.org/licenses/by/4.0/), which permits unrestricted use, distribution, and reproduction in any medium, provided the original author and source are credited

8 Open Adobe® Illustrator®.

9 Select: **Create new** > choose size of preference

10 Set the workspace to tracing: **Window** > **Workspace** > **Essentials Classic**

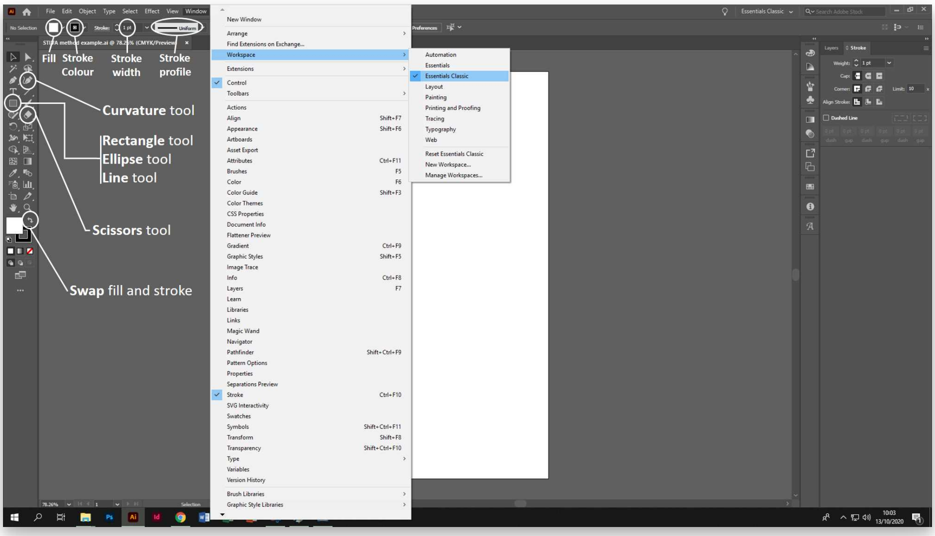

Snapshot of configured workscape, with labelled positions of the basic tools and functions which will be needed throughout the protocol.

11 Set up the layers that will be used using the **Create new layer** button.

11.1 Create the following layers in ascending (bottom to top) order: "background", "base photograph", "cortex" (only if present), "outline", "scars borders", "ripples", "symbols" (only if present), and "scale".

Citation: Jacopo Niccolo Cerasoni (04/19/2021). Stone Tools Illustrations with Vector Art: The 'STIVA' Method. https://dx.doi.org/10.17504/protocols.io.bubqnsmw

This is an open access protocol distributed under the terms of the **Creative Commons Attribution License** (https://creativecommons.org/licenses/by/4.0/), which permits unrestricted use, distribution, and reproduction in any medium, provided the original author and source are credited

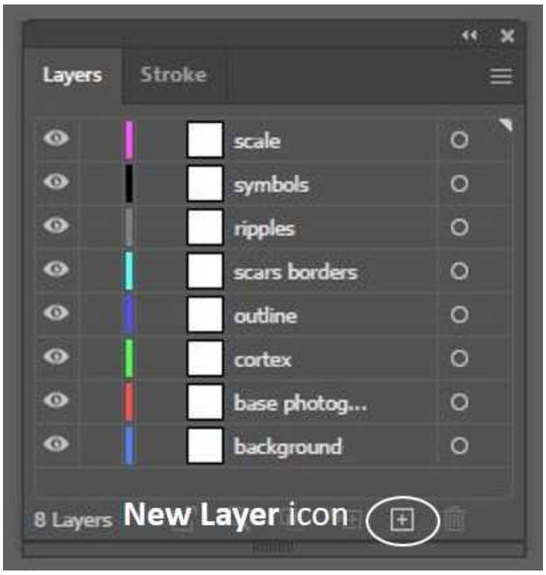

Layers tab with position of **New Layer** icon.

Upload Photograph

12 Select the layer "base photograph", making sure the layer is highlighted in blue in the **Layers** tab.

12.1 Insert (import, drag or copy and paste) the previously selected photograph onto the layer.

13 Lock layer “base photograph” by clicking the **lock** icon.

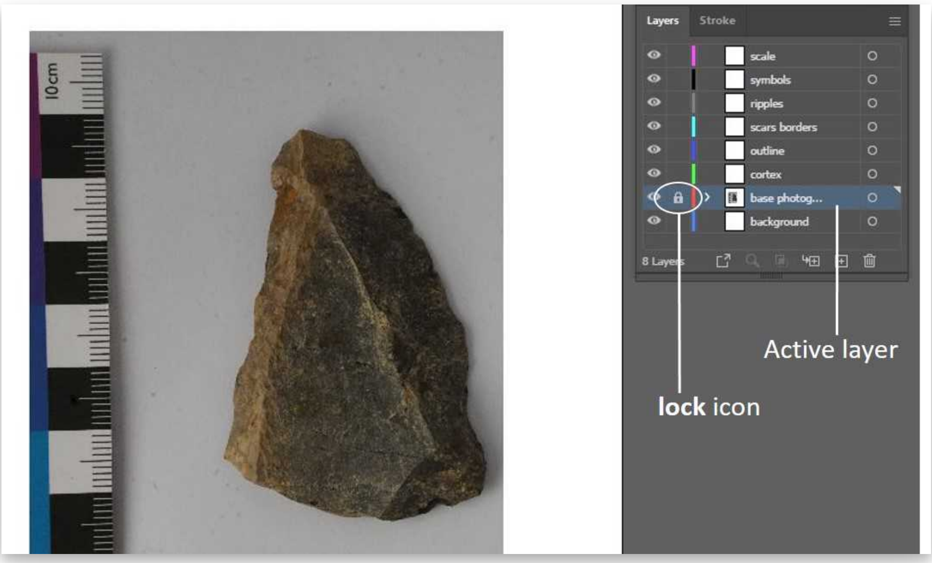

Snapshot of artboard after steps *12* and *13.*

Citation: Jacopo Niccolo Cerasoni (04/19/2021). Stone Tools Illustrations with Vector Art: The 'STIVA' Method. https://dx.doi.org/10.17504/protocols.io.bubqnsmw

This is an open access protocol distributed under the terms of the **Creative Commons Attribution License** (https://creativecommons.org/licenses/by/4.0/), which permits unrestricted use, distribution, and reproduction in any medium, provided the original author and source are credited

Add Scale

14 Decide how long the scale will be (common scale lengths are: 1cm, 2cm, 5cm, 10cm and 20cm).

15 Trace the selected length scale over the "base photograph" scale with the **Rectangle Tool.**

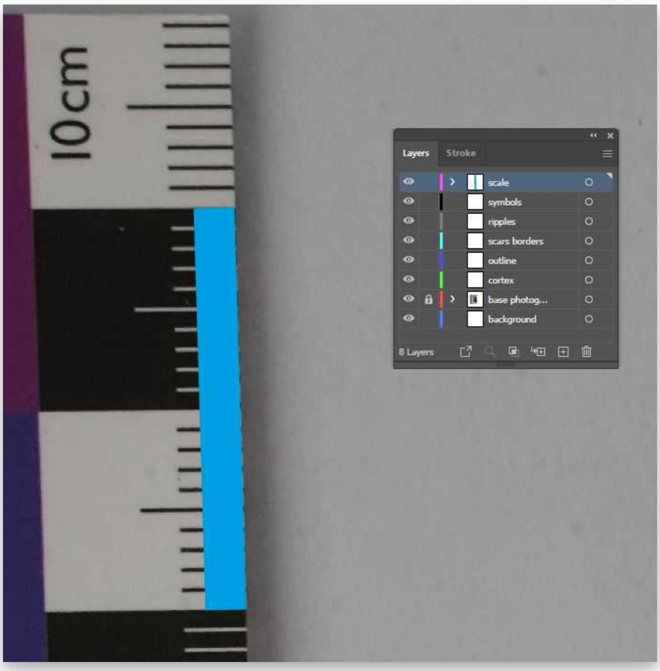

Snapshot of artboard of scale creation.

Illustrate Artefact Outline

16 Use **Curvature Tool** to trace the outline of the artefact as a closed path. This action should be done while at a high level of magnification and using a medium to thin line stroke thickness, so to maintain as much detail as possible.

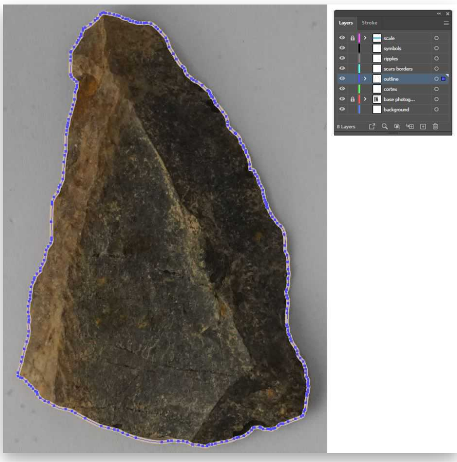

Completed "outline" layer showing drawn anchor points.

Citation: Jacopo Niccolo Cerasoni (04/19/2021). Stone Tools Illustrations with Vector Art: The 'STIVA' Method. https://dx.doi.org/10.17504/protocols.io.bubqnsmw

This is an open access protocol distributed under the terms of the **Creative Commons Attribution License** (https://creativecommons.org/licenses/by/4.0/), which permits unrestricted use, distribution, and reproduction in any medium, provided the original author and source are credited

17 Lock layer "outline".

Illustrate Scars Borders

18 Use **Curvature Tool** to trace the scars borders of the artefacts as open paths. Set the thickness of the paths at the same thickness of the artefact outline.

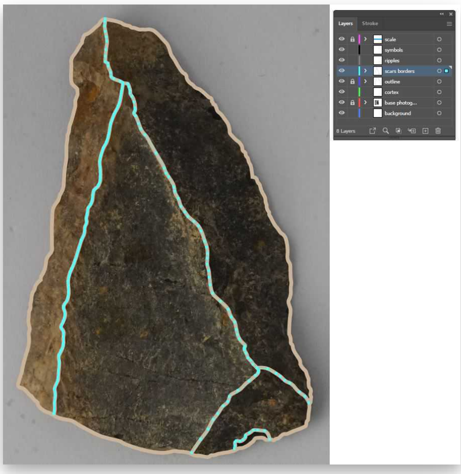

Completed "scars borders" layer showing drawn anchor points.

19 Whenever the scars borders are difficult to distinguish, view the original artefact under lateral light for direct observation of ambiguous areas.

20 Lock layer “scars borders”.

Illustrate Ripples

21 Select a scar, view it at different magnifications, and observe its ripple direction, depth and texture variations.

22 Use **Curvature Tool** to trace one or more reference ripples, which will be traced over any visible ripple marks found on the “base photograph”.

Citation: Jacopo Niccolo Cerasoni (04/19/2021). Stone Tools Illustrations with Vector Art: The 'STIVA' Method. https://dx.doi.org/10.17504/protocols.io.bubqnsmw

This is an open access protocol distributed under the terms of the **Creative Commons Attribution License** (https://creativecommons.org/licenses/by/4.0/), which permits unrestricted use, distribution, and reproduction in any medium, provided the original author and source are credited

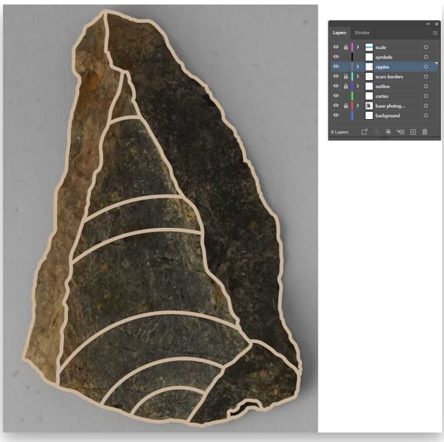

Completed reference ripples as explained in step *22*.

22.1 The number of reference ripples will depend upon the length of the scar (e.g. a small retouch scar will need one, a long fluting scar will need 5 or more).

22.2 Start on one side of the ripple mark from the scar border and draw an open path to the opposite scar border. Ripples should be composed by only 3 anchor points (nodes); one at each edge, and one along the path.

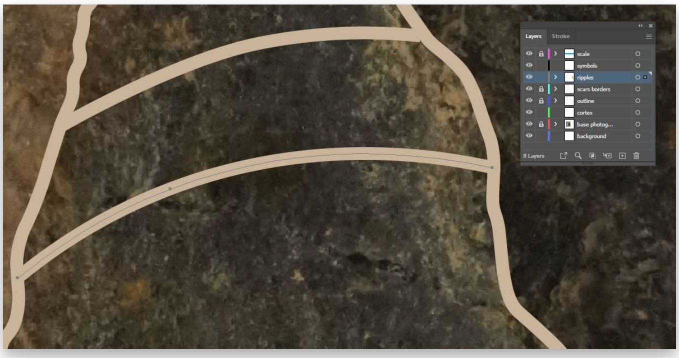

Example of ripple construction. Note the selected open path is constructed using three anchor points.

22.3 Important note: when drawing the open path ripple, the direction (first to last anchor) should follow the desired direction of the final ripple, as this will be important for step *27*.

22.4 If no specific ripple marks are visible on the "base photograph", directly observe the artefact under lateral lighting for direct observation of orientation and depth of ripple marks.

22.5 If it is not possible to access the artefact directly, view the previously unselected photographs taken with different lighting orientations to discern any identifiable ripple marks.

Citation: Jacopo Niccolo Cerasoni (04/19/2021). Stone Tools Illustrations with Vector Art: The 'STIVA' Method. https://dx.doi.org/10.17504/protocols.io.bubqnsmw

This is an open access protocol distributed under the terms of the **Creative Commons Attribution License** (https://creativecommons.org/licenses/by/4.0/), which permits unrestricted use, distribution, and reproduction in any medium, provided the original author and source are credited

23 Use **Curvature Tool** to draw more ripples, using the same path construction and thickness as in step *22*.

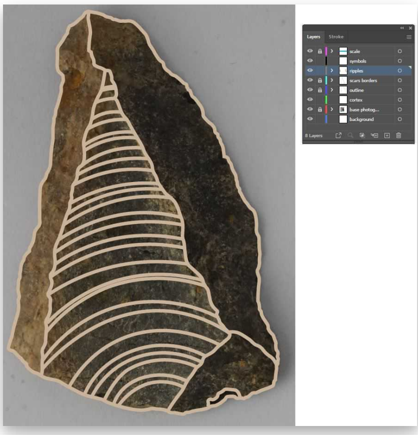

Completed ripples as explained in step *24*.

23.1 The density of ripples and distance between them will be dependent on the variation in depth of the scar surface (e.g. very flat scar surface will have distant and sporadic ripples, very concave scars and hinge terminations will have dense and close ripples).

24 Repeat steps *21-24* for all the remaining scars.

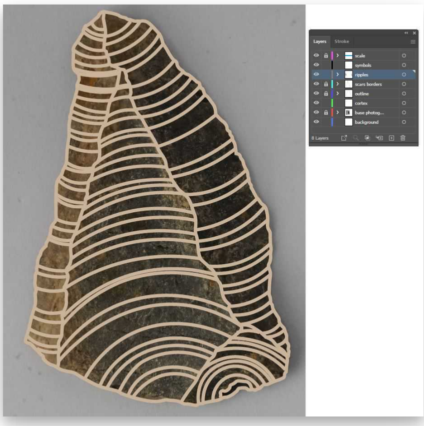

Completed ripples on the whole lithic surface as explained in step *21-25*.

25 Simplify ripples by selecting them, and then select:

Object > **Path** > **Simplify...** > Set indicator to maximum

Cut Ripples Marks To Length

26 Use **Scissors Tool** to cut ripple to length. This step will give dimensionality to the flaked scars, with the intent to inform the viewer on the depth, directionality and topography of the scar.

Note: Keep in mind the open path direction that was set in step *22.4*.

Citation: Jacopo Niccolo Cerasoni (04/19/2021). Stone Tools Illustrations with Vector Art: The 'STIVA' Method. https://dx.doi.org/10.17504/protocols.io.bubqnsmw

This is an open access protocol distributed under the terms of the **Creative Commons Attribution License** (https://creativecommons.org/licenses/by/4.0/), which permits unrestricted use, distribution, and reproduction in any medium, provided the original author and source are credited

26.1 To properly cut the ripple marks, the surface has to be assessed and identified, so to distinguish areas of the scars which are flat and regular versus areas of the scars which are deep and irregular.

When cutting a ripple mark, the following rules should be applied:

- If the area where the ripple mark is located is ***flat and/or regular***, then the ripple mark will be ***long*** (cut a short portion of the ripple mark)

- If the area where the ripple mark is located is ***deep and/or steep and/or irregular***, then the ripple mark will be ***short*** (cut a long portion of the ripple mark)

26.2 To increase efficiency, cut a path at the desired point along the ripple path and press <Del> or <Canc> button on keyboard right after. This should automatically delete the unwanted path section without having to change tool for each ripple.

27 Repeat step *27* for all ripple marks.

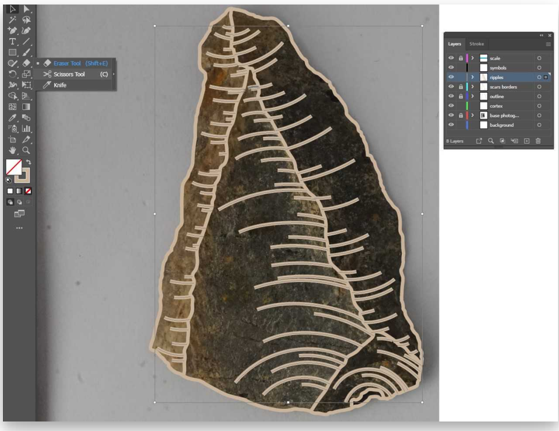

Cut ripples as explained in steps *27* and *28*.

Feather Ripples

28 Select all ripples, and change profile to **width profile 4**.

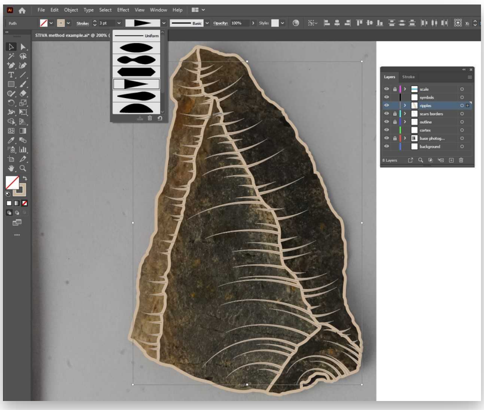

Feathered ripples as explained in step *29*. Note, the **width profile 4** option is selected on the upper functions tab.

Citation: Jacopo Niccolo Cerasoni (04/19/2021). Stone Tools Illustrations with Vector Art: The 'STIVA' Method. https://dx.doi.org/10.17504/protocols.io.bubqnsmw

This is an open access protocol distributed under the terms of the **Creative Commons Attribution License** (https://creativecommons.org/licenses/by/4.0/), which permits unrestricted use, distribution, and reproduction in any medium, provided the original author and source are credited

29 Lock layer “ripples”.

Adjust Stroke Widths

30 Adjust stroke width of layers: “outline”, “scar borders”, and “ripples”.

To adjust the stroke width, unlock the layer, select all paths within layer by pressing the “select all” indicator, and adjust stroke width.

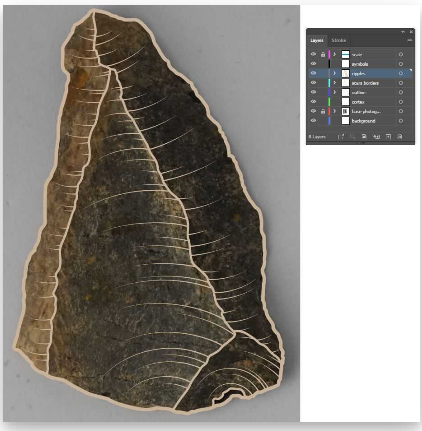

View of illustration after adjusting "outline", "scars borders" and "ripples" stroke widths as explained in step *31*.

30.1 The following stroke width ratio is recommended: “outline” - 3:3 , “scar borders” - 2:3 , “ripples” - 1:3

31 Lock layers “outline”, “scar borders”, and “ripples”.

Draw Cortex

32 Before drawing the cortex, **Basic Graphic_Dots** fill has to be activated as a stroke.

32.1 Draw an open path with **Curvature Tool**. Set fill to any **Basic Graphic_Dots** fill. To do so: Click **Fill** drop down icon > **Swatch Libraries menu** > **Patterns** > **Graphic Patterns** > **Basic Graphic_Dots** > choose dotted fill of choice.

Citation: Jacopo Niccolo Cerasoni (04/19/2021). Stone Tools Illustrations with Vector Art: The 'STIVA' Method. https://dx.doi.org/10.17504/protocols.io.bubqnsmw

This is an open access protocol distributed under the terms of the **Creative Commons Attribution License** (https://creativecommons.org/licenses/by/4.0/), which permits unrestricted use, distribution, and reproduction in any medium, provided the original author and source are credited

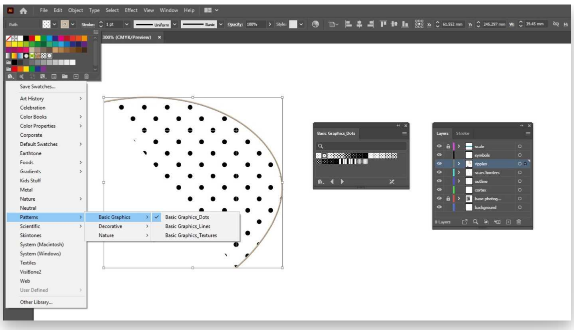

Snapshot of step *33.1*.

32.2 Click **Swap Fill and Stroke** button.

32.3 Delete open path.

33 Use **Curvature Tool** or **Pen Tool** to trace the cortex outline as a closed path tracing carefully the "outline" and "scar borders" paths that border the cortex area.

34 Create new dot-pattern brush.

34.1 Select: **Window** > **Brushes** > **New Brush** > **Pattern Brush**

34.2 Name your new brush.

34.3 Click each **tile** drop down menu and select the doted pattern of choice (generally **10 dpi 10-30%** are recommended).

Citation: Jacopo Niccolo Cerasoni (04/19/2021). Stone Tools Illustrations with Vector Art: The 'STIVA' Method. https://dx.doi.org/10.17504/protocols.io.bubqnsmw

This is an open access protocol distributed under the terms of the **Creative Commons Attribution License** (https://creativecommons.org/licenses/by/4.0/), which permits unrestricted use, distribution, and reproduction in any medium, provided the original author and source are credited

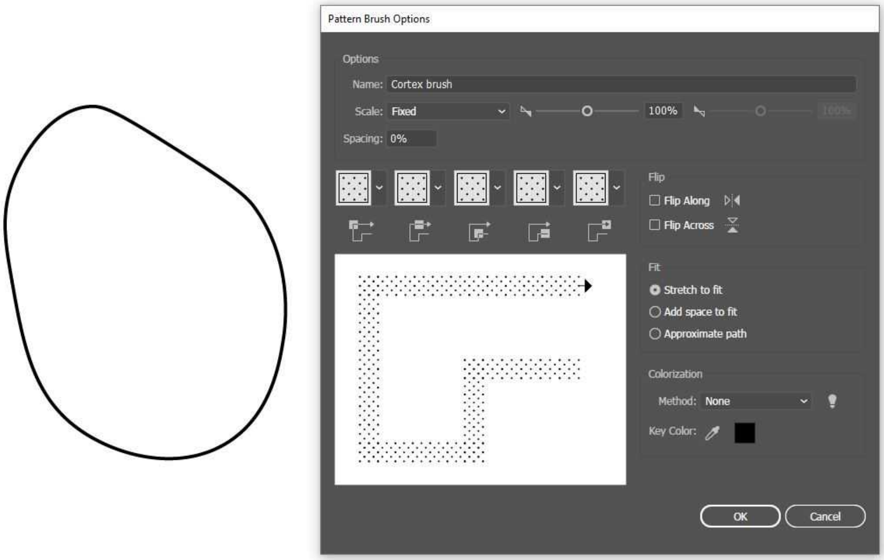

Snapshot of step *35.3*.

34.4 Once all tiles have been set, the preview window will show what the final brush will look like. Click **OK**.

35 Use **Paintbrush Tool** set on the newly created pattern brush to fill in the cortex outline.

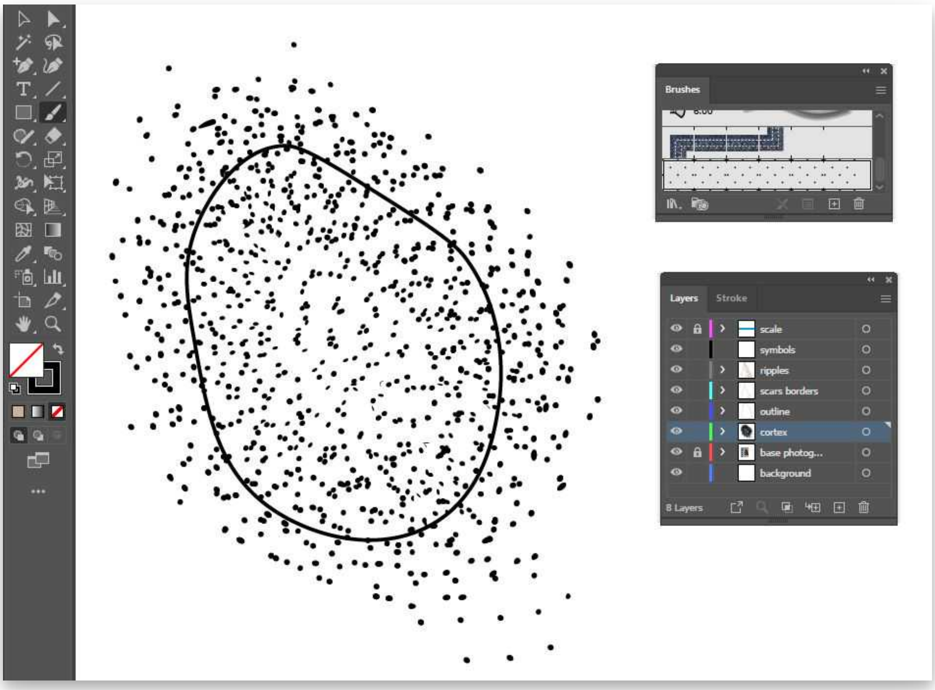

Completed brush strokes as explained in step *36*.

Citation: Jacopo Niccolo Cerasoni (04/19/2021). Stone Tools Illustrations with Vector Art: The 'STIVA' Method. https://dx.doi.org/10.17504/protocols.io.bubqnsmw

This is an open access protocol distributed under the terms of the **Creative Commons Attribution License** (https://creativecommons.org/licenses/by/4.0/), which permits unrestricted use, distribution, and reproduction in any medium, provided the original author and source are credited

35.1 The brush can be used to regularly fill in the cortex area will parallel strokes or used to characterise texture and irregularities.

35.2 The brush strokes can overlap and be drawn outside the cortex outline, attention should only be given to the fill within the cortex outline.

36 Select all the brush strokes from step *36*, making sure to not select the cortex outline.

36.1 <right click> the selected paths, and **group**.

37 Select the grouped brush paths, then:

<right click> > **Arrange** > **Bring to Back**

38 Select the grouped brush paths and the cortex outline.

38.1 Click <right click> > **Make Clipping Mask**

38.2 The final result should show only the cortex area filled in with the desired pattern.

Citation: Jacopo Niccolo Cerasoni (04/19/2021). Stone Tools Illustrations with Vector Art: The 'STIVA' Method. https://dx.doi.org/10.17504/protocols.io.bubqnsmw

This is an open access protocol distributed under the terms of the **Creative Commons Attribution License** (https://creativecommons.org/licenses/by/4.0/), which permits unrestricted use, distribution, and reproduction in any medium, provided the original author and source are credited

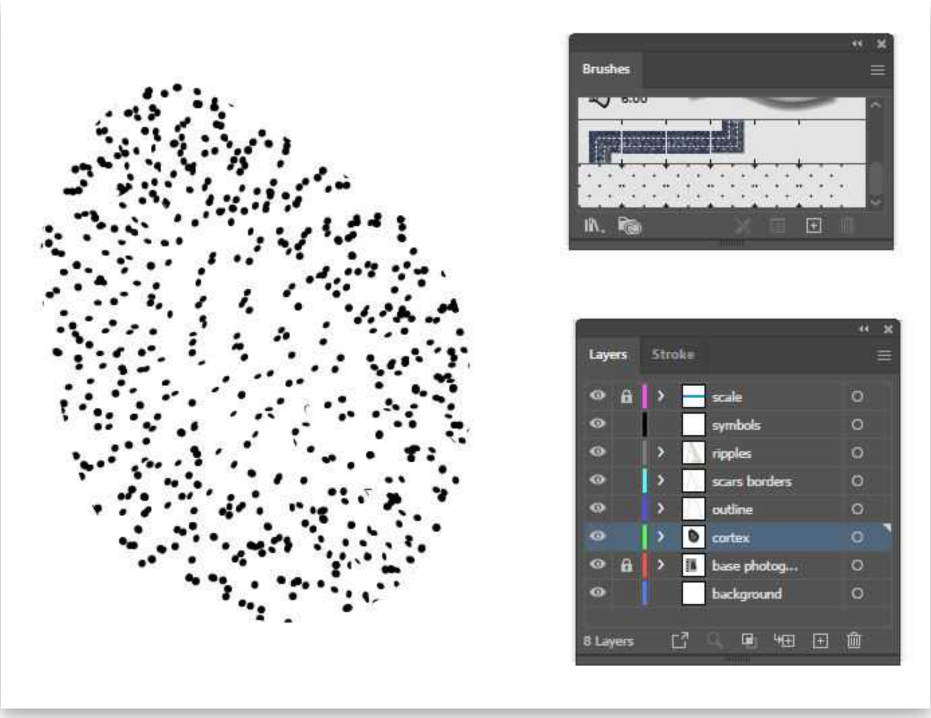

Final result for cortex illustration as explained in steps *33-39*.

39 Lock layer “cortex”.

Add symbols

40 Any further symbols (e.g. percussion point, retouch extension, wear extension, burin) can be added using a variety of vectorial tools such (e.g. **Rectangle Tool**, **Ellipse Tool**, **Line Tool**, **Curvature Tool**, **Pen Tool**)

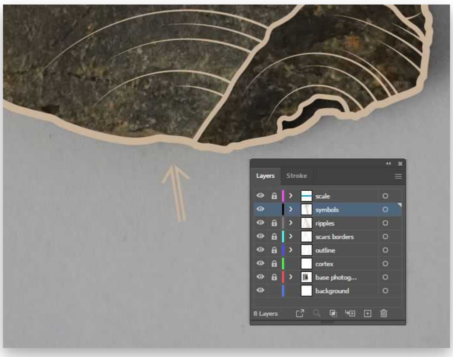

Example of arrow showing position and directionality of percussion point

41 Lock layer “symbols”.

Create Artefact Composite

Citation: Jacopo Niccolo Cerasoni (04/19/2021). Stone Tools Illustrations with Vector Art: The 'STIVA' Method. https://dx.doi.org/10.17504/protocols.io.bubqnsmw

This is an open access protocol distributed under the terms of the **Creative Commons Attribution License** (https://creativecommons.org/licenses/by/4.0/), which permits unrestricted use, distribution, and reproduction in any medium, provided the original author and source are credited

42 Repeat processes *12-42* to illustrate the remaining artefact sides.

43 Remove "base photographs" from all the illustrations.

43.1 Use **Rectangle Tool** to draw a background with a colour of choice onto "background" layer.

43.2 Modify **stroke colour** of the figures to match the background and offer good visual contrast.

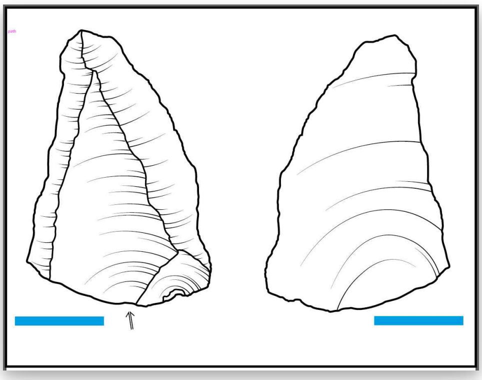

Composition with corrected colour pattern as explained in step *44*.

44 If the figures are not at the same scale, rescale them separately so to make them regular. Use the scale bars (drawn during step *15*) and **rulers** (<Ctrl>/<Cmd> + <R>) to double check accuracy.

44.1 Once complete, delete any repeating duplicate scale.

45 Move and arrange the different illustrations so to create a well ordered composition. Use the **rulers** and **guide** to make sure that the intervals between the figures are regular.

Citation: Jacopo Niccolo Cerasoni (04/19/2021). Stone Tools Illustrations with Vector Art: The 'STIVA' Method. https://dx.doi.org/10.17504/protocols.io.bubqnsmw

This is an open access protocol distributed under the terms of the **Creative Commons Attribution License** (https://creativecommons.org/licenses/by/4.0/), which permits unrestricted use, distribution, and reproduction in any medium, provided the original author and source are credited

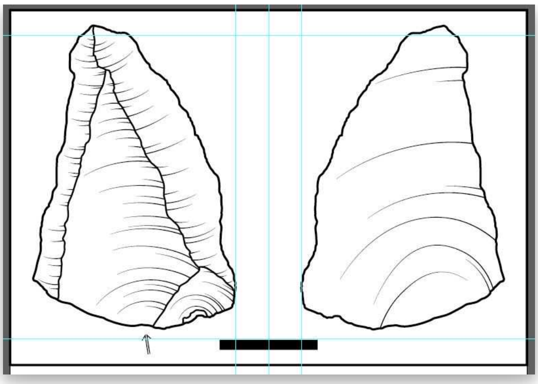

Snapshot of the figure composition ordering through the use of **guides** (blue lines) as explained in step *46*.

Crop Artboard

46 Once the composition is complete, resize the artboard:

File > **Document Setup…** > **Edit Artboard** > Crop artboard to desired size

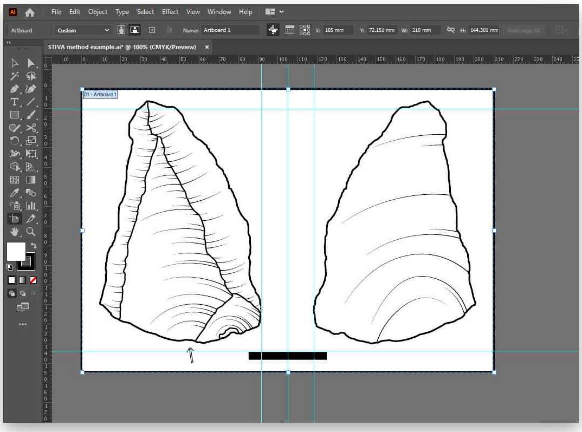

Completed artboard resizing as explained in step *47*.

Finishing Touches

47 Double check all the paths, making sure there are no protruding nodes, and that all strokes are overlapping correctly.

48 Add any final object that is required, creating new layers for each.

48.1 Use **Line Tool** or **Rectangle Tool** to connect the different illustrations, and add them in new layer "connectors".

48.2 Use **Type Tool** to add text labels for the scale and for any other information that wants to be added (e.g. specimen number, features, figure numbering) in new layer "text".

Citation: Jacopo Niccolo Cerasoni (04/19/2021). Stone Tools Illustrations with Vector Art: The 'STIVA' Method. https://dx.doi.org/10.17504/protocols.io.bubqnsmw

This is an open access protocol distributed under the terms of the **Creative Commons Attribution License** (https://creativecommons.org/licenses/by/4.0/), which permits unrestricted use, distribution, and reproduction in any medium, provided the original author and source are credited

49 Your final illustration is complete.

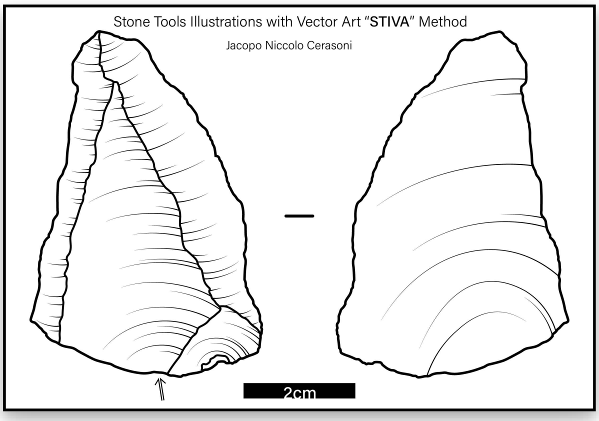

Export Final Figure

50 On Adobe® Illustrator® a variety of export formats can be selected. The most common are:

1. .PDF (**File** > **Save as...** > **Save as type:** > **Adobe PDF**)

2. .JPG (**File** > **Export** > **Export as...** > **Save as type**: > **JPEG**)

3. .TIF (**File** > **Export** > **Export as...** > **Save as type:** > **TIFF**)

Citation: Jacopo Niccolo Cerasoni (04/19/2021). Stone Tools Illustrations with Vector Art: The 'STIVA' Method. https://dx.doi.org/10.17504/protocols.io.bubqnsmw

This is an open access protocol distributed under the terms of the **Creative Commons Attribution License** (https://creativecommons.org/licenses/by/4.0/), which permits unrestricted use, distribution, and reproduction in any medium, provided the original author and source are credited
