# Supplementary material for: Vectorial application for the illustration of archaeological lithic artefacts using the “Stone Tools Illustrations with Vector Art” (STIVA) Method
Source: PLoS One. 2021 May 11;16(5):e0251466. doi: 10.1371/journal.pone.0251466 (PMC8112888; doi:10.1371/journal.pone.0251466)
Supplement: Attachment — Submitted filename: Response to Reviewers.docx [file pone.0251466.s002.docx]

Reviewer’s Comments

Author’s Response

Changes Made

Reviewer #1:

This paper presents a step-by-step guide outlining a novel method of illustrating lithic artifacts using Adobe Illustrator (STIVA Method). The guide is clear and easy to follow, allowing a wide range of archaeologists to digitally draw their own lithic artifacts. The capacity to illustrate in this manner is not new, but it is useful in that it makes people aware of the full functionality of programs such as Adobe Illustrator and makes it accessible to people who may not be familiar with the program. It also provides a framework for others to build upon to illustrate other objects found in archaeological contexts.

While the step-by-step guide is nicely put together (see minor comments below), the remainder of the manuscript needs a bit of editing and elaboration.

The Introduction would be greatly improved if the author added further discussion of where the STIVA method fits in with other ways of representing lithic artifacts. For example, the author juxtaposes photography with hand illustrations, arguing that photography is often not detailed enough to capture the relevant nuances present on lithic artifacts. However, morphometrics and 3-D scanning are a growing field, and this deserves further attention. A brief discussion of the advantages and disadvantages of various methods of illustration and representation, replete with expanded references, would be welcome.

Thank you for these comments. Further discussion in the Introduction regarding other methods of lithic illustrations is very welcome and appreciated by the author. Other methods (i.e. photogrammetry and 3-D scanning), together with their advantages and disadvantages, have been included. The bibliography has been expanded to include more references which exhibit and discuss the use of photography, photogrammetry and 3-D scanning in lithic representation and studies.

The Introduction, from line 64 to line 73, has been reworded and rewritten to clarify the juxtaposition of photography with hand illustration.

Two paragraphs have been added in the Introduction, presenting and elaborating on the applications and use of photogrammetry and 3-D scanning.

The manuscript also suffers from a number of grammatical and wording issues, and I would suggest systematic copyediting to improve its flow and readability.

Thank you for this comment. Systematic copyediting was carried out by an external professional to solve all grammatical and wording issues.

Specific Comments:

• Line 47: Divulgation is perhaps the wrong word. The first sentence of the introduction should be reworded.

Thank you for this comment. The first sentence has been reworded as shown below:

Archaeological research involves the study of past human activity, behaviour, and interaction with the environment.

• Line 57-58: The following is too informal: “…has been used for the longest time in human prehistory.”

Thank you for this comment. The sentence has been removed.

• Line 102: Change “whilst” to “while”

Thank you for this comment. “Whilst” has been changed to “while”.

• Can steps 22 and 23 be combined?

A new version of the protocol has been created (v.2), where steps 22 and 23 have been combined.

• Based on your discussion in step 27, the cutting of ripple marks seems to be rather subjective. This should be made clearer in step 27.1, perhaps stating why the cutting of ripple marks is needed. Any other suggestions on the best way to do this would also be welcome.

Following the reviewer’s comment, step 26 (27 in v.1), has been modified as follows:

Step 26

Use **Scissors Tool** to cut ripple to length. This step will give dimensionality to the flaked scars, with the intent to inform the viewer on the depth, directionality and topography of the scar.

Note: Keep in mind the open path direction that was set in step *22.4*.

Sub-step 26.1

To properly cut the ripple marks, the surface has to be assessed and identified, so to distinguish areas of the scars which are flat and regular versus areas of the scars which are deep and irregular.

When cutting a ripple mark, the following rules should be applied:

- If the area where the ripple mark is located is ***flat and/or regular***, then the ripple mark will be ***long*** (cut a short portion of the ripple mark)
- If the area where the ripple mark is located is ***deep and/or steep and/or irregular***, then the ripple mark will be ***short***

Sub-step 26.2

To increase efficiency, cut a path at the desired point along the ripple path and press <Del> or <Canc> button on keyboard right after. This should automatically delete the unwanted path section without having to change tool for each ripple.
